# A Versatile, Machine-Learning-Enhanced RF Spectral Sensor for Developing a Trunk Hydration Monitoring System in Smart Agriculture

**DOI:** 10.3390/s24196199

**Published:** 2024-09-25

**Authors:** Oumaima Afif, Leonardo Franceschelli, Eleonora Iaccheri, Simone Trovarello, Alessandra Di Florio Di Renzo, Luigi Ragni, Alessandra Costanzo, Marco Tartagni

**Affiliations:** 1 Department of Electrical, Electronic and Information Engineering, Guglielmo Marconi-University of Bologna, Via Dell’Università, 50, 47521 Cesena, Italy; leonar.franceschell2@unibo.it (L.F.); simone.trovarello2@unibo.it (S.T.); alessandra.diflorio3@unibo.it (A.D.F.D.R.); alessandra.costanzo@unibo.it (A.C.); marco.tartagni@unibo.it (M.T.); 2 Department of Agricultural and Food Sciences, Alma Mater Studiorum, University of Bologna, Piazza Goidanich 60, 47521 Cesena, Italy; eleonora.iaccheri4@unibo.it (E.I.); luigi.ragni@unibo.it (L.R.); 3 Interdepartmental Center for Industrial Agri-Food Research, University of Bologna, Via Q. Bucci 336, 47521 Cesena, Italy

**Keywords:** microwave sensors, NanoVNA, Raspberry Pi, precision agriculture, machine learning

## Abstract

This paper comprehensively explores the development of a standalone and compact microwave sensing system tailored for automated radio frequency (RF) scattered parameter acquisitions. Coupled with an emitting RF device (antenna, resonator, open waveguide), the system could be used for non-invasive monitoring of external matter or latent environmental variables. Central to this design is the integration of a NanoVNA and a Raspberry Pi Zero W platform, allowing easy recording of S-parameters (scattering parameters) in the range of the 50 kHz–4.4 GHz frequency band. Noteworthy features include dual recording modes, manual for on-demand acquisitions and automatic for scheduled data collection, powered seamlessly by a single battery source. Thanks to the flexibility of the system’s architecture, which embeds a Linux operating system, we can easily embed machine learning (ML) algorithms and predictive models for information detection. As a case study, the potential application of the integrated sensor system with an RF patch antenna is explored in the context of greenwood hydration detection within the field of smart agriculture. This innovative system enables non-invasive monitoring of wood hydration levels by analyzing scattering parameters (S-parameters). These S-parameters are then processed using ML techniques to automate the monitoring process, enabling real-time and predictive analysis of moisture levels.

## 1. Introduction

### 1.1. State of the Art

The advancement of radio frequency (RF) integrated circuits (ICs) is the primary driving force behind the current rapid progress in portable and low-cost vector network analyzer (VNA) technology [1]. Since the inception of the first devices in the 1950s, VNAs have undergone continuous enhancement in terms of accuracy, speed, miniaturization, and functionality. These advancements have solidified their status as indispensable instruments for research, development, manufacturing, and testing purposes [2]. Notably, VNAs serve as the primary measurement tools utilized for characterizing circuits and devices across radio, microwave, millimeter-wave, and submillimeter-wave frequencies [3,4]. Indeed, VNAs have become highly sought-after instruments that extend beyond the domain of electronic and telecommunications engineering. Emerging applications of VNAs encompass the measurement of various materials, biological samples, plants, and soils [5,6,7,8,9,10,11,12,13]. In fields like dielectric spectroscopy, when coupled with appropriate sensors, the VNA offers invaluable capabilities [14,15]. When coupled with one or more antennas, it transforms into a radar system, capable of detecting material flaws imperceptible to the naked eye, even without the use of X-rays [16]. However, the utilization of VNAs typically remains limited to stationary scenarios because of their size, weight, and high cost [17]. Consequently, there exists a growing demand for a compact, affordable, and portable VNA with sufficient accuracy and functionality. Such a device is regarded as a promising noninvasive measurement technique applicable across a wide range of fields [18]. However, the initial efforts to develop cost-effective functional VNAs were primarily targeted at the amateur radio market, focusing on the HF and VHF portions of the spectrum [19]. Another well-known series of devices is the nanoVNA series, which has given rise to numerous variants [20]. A separate VNA, named nanoVNA v2, has recently emerged and developed independently from the original one. The latter represents a portable yet high-performance tool. This compact handheld device operates as a battery-powered, self-contained LCD gadget [21]. In its initial development stages, the NanoVNA was designed to function within the 50 kHz–300 MHz frequency range. The operation of this nano-analyzer’s mixer SA612A necessitates a 5 V power supply, which cannot be directly sourced from the battery. Therefore, an initial version of the NanoVNA required a USB power supply for operation. For this reason, Hugen redesigned the NanoVNA based on the edy555 schematic and incorporated a DC-DC circuit, enabling it to operate independently [22]. A new version of the NanoVNA called NanoVNA V2 (S-A-A-2) has been released, which can operate within a frequency range of 50 kHz–4.4 GHz [23]. Like [24], one of the few studies that have proposed a method that utilizes a Raspberry Pi to act as an interface between a VNA and a universal arm robot to perform automated measurements, the focus of this study is on robotics. The system considered in that study, consisting of a VNA, an antenna horn, and a Raspberry Pi, is not portable. Nevertheless, there is always a need for improvement of the NanoVNA by employing solutions for data-acquisition-managed local processing, and miniaturizing it by removing its LCD screen to make it fully wearable [25].

As far as the application chosen in this paper is concerned, assessing wood’s physical attributes without causing damage is crucial in contemporary orchard production procedures [26]. The amount of water transpired by a plant is an important factor for irrigation management and the study of plant–water relations. Measuring the volumetric sap flow rate in plant stems provides a method for estimating transpiration, which is essential in precision agriculture. It is known that water stress and sap flow measurements are the most sought feature in irrigation management; however, they are complex to implement in the field. Therefore, the evaluation of trunk moisture content (MC), even if it misses some information, could be a faster and more reliable technique to fulfill the main requirements. Two primary methods for determining the MC of wood can be identified. The dielectric characteristics of wood are influenced by factors such as density, MC, temperature, and frequency [27]. The first involves direct measurements, in which the MC is assessed through oven drying, which is considered to be the most accurate method for determining the MC of materials according to standardized processes (see EN ISO 12570 [28]). However, in commercial woodworking practice as well as in the field, there is a pressing need for immediate and highly accurate determination of the MC of materials [29]. Indirect measurement techniques are indeed quick in determining the MC [30]. These technologies encompass multiple methods, such as electrical resistance measurements [31], acoustics [32], thermal [33,34], near-infrared spectroscopy (NIR) [35], and RF [36]. The latter method fulfills all the desirable criteria of being non-destructive, non-contact, accurate, robust, and rapid simultaneously. It is typically useful for monitoring purposes, but its current application might be hindered by the relatively high cost of the measurement equipment it requires, and it seems that there is still a need for more affordable, low-cost, and stand-alone techniques for monitoring wood MC. Sap flow evaluation by thermal methods seems to be the most accurate and relies on detecting temperature changes in the sap using thermocouples placed at locations distant from a heat source. However, even if very useful in agricultural research, it is time consuming in the field, as it requires drilling into the trunk or the precise attachment of sensors and actuators. For these reasons, the proposed approach is promising since it is non-invasive and does not require trunk modification. It is easy to install in a shorter timeframe in a working field scenario, allowing for the monitoring of many trees for irrigation purposes.

### 1.2. Proposed Approach and Application

**Figure 1:**
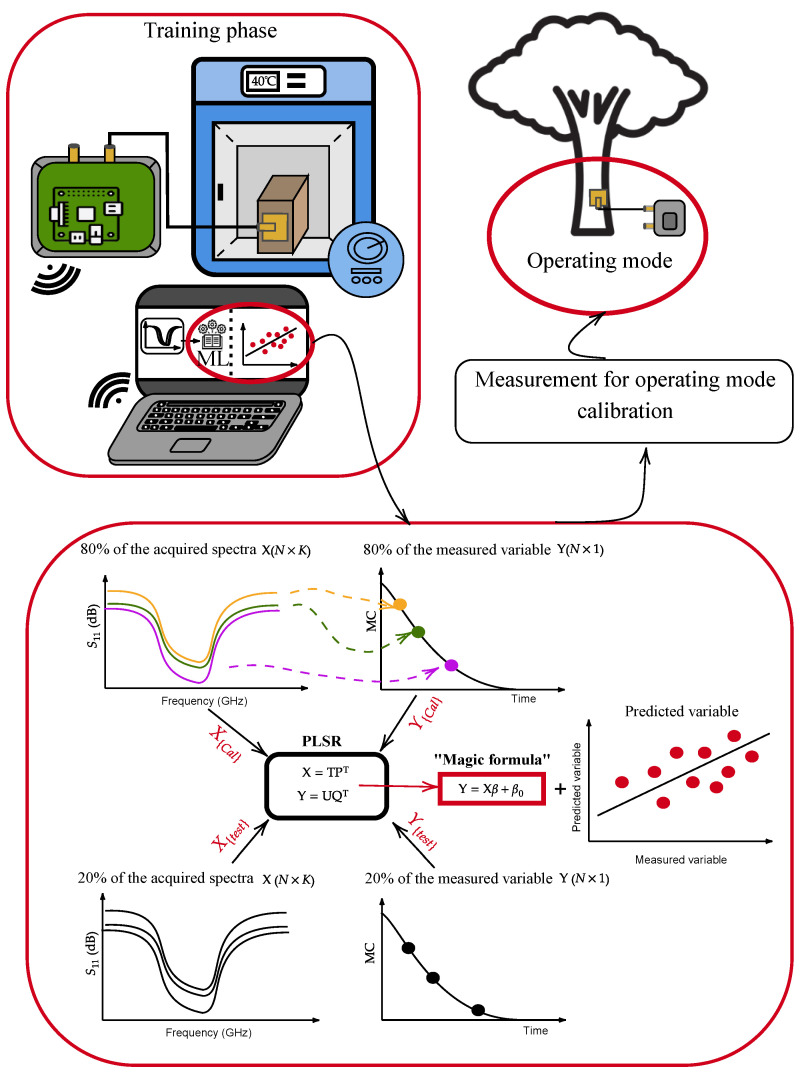
Overview of the proposed system and application.

Following the needs of the application and the drawbacks of currently used techniques, we developed a patch-type antenna spectral sensor to be installed on the upper horizontal surface of greenwood samples to capture the RF spectra of the microwave signals reflected by the wood. Since the device is noninvasive, it could be easily applied, removed, and replicated in multiple devices for orchard monitoring. The approach is shown in Figure 1. To demonstrate the feasibility of the technique, thanks to the versatility of the proposed device, we performed long-term acquisitions of scattering parameter spectra of a greenwood trunk chop forced to progressively dry in a climate chamber. Then, we performed ML algorithms on the acquired spectra to understand the correlation with hydration values.

The device is based on a versatile standalone and miniature system using a NanoVNA as an RF sensor frontend working in both manual and automatic modes. To accomplish that, we used a Raspberry Pi Zero as a control system and fused the two systems using a custom printed circuit board (PCB) board. It facilitates powering both components using a singular battery source and automatically capturing spectra. Data acquisition is performed through a Python function saved in a Raspberry Pi Zero W, which is also used for storing the data and controlling all the parameters necessary for the acquisition. The features of the presented system represent a first example in the integration of a low-cost microwave-based platform for precise and automated S-parameter measurements. The MC detection in the wood sample is carried out by adopting a patch antenna, operating at 2.45 GHz, as the sensing element. Patch antennas are widely exploited in RF applications for their low profile, low cost, and ease of manufacturing. Although patch antennas are commonly used in wireless links [37,38], vehicle positioning [39], energy harvesting/WPT [40,41,42], and biomedical applications [43], they are not extensively exploited for sensing applications. In [44], a first attempt to use a patch antenna as a sensing element for MC detection is presented. Common RF-based sensors exploit near-field resonators such as split-ring resonators (SRRs) and complementary split-ring resonators (CSRRs) [45]. Although the latter offer high performance in terms of quality factors, they have low penetration thicknesses, making them unsuitable in solutions where the chemical and/or physical property to be analyzed resides in the innermost layers of the material under test. Therefore, a patch-type antenna, operating in the near-field radiative region, is used as an MC detector. However, a bare automatic VNA could hardly be considered a sensor of some physical entity because spectra raw data hide the sought information [46]. For this reason, a machine learning (ML) post-processing technique should be employed to extract sensitive variables. Therefore, the acquired spectra were collected into a dataset, and an ML predictive model was derived. The latter could then be used in operating mode to monitor, in real time and in a real scenario, the amount of water flowing in a trunk to understand watering needs.

The main contributions presented in this paper are twofold. First, from the architectural implementation viewpoint we will present: (i) a compact hardware (HW) integration between a Raspberry Pi Zero W, a single-board computer (SBC) and a NanoVNA V2 (S-A-A-2) operating in the range of 50 KHz–4.4 GHz [23,47,48], using specific PCB and case design; (ii) a data acquisition procedure based on Python scripts for manual and automated S-parameter acquisition and data storage. Secondly, from the case study demonstration point of view, we will show (i) the coupling with a patch antenna for hydration sensing of a cut wood trunk; (ii) a long-term automation acquisition of the S-parameter spectra of fresh wood dehydrated in a drying oven; (iii) an ML predictive model (embedded in the system) for hydration quantitative evaluation. The structure of this paper is as follows: Section 2 presents the main architecture and materials. Section 3 introduces the modes of operation. Section 4 is dedicated to a case study, addressed to the agriculture field, applying the new PCB. Finally, Section 5 provides a summary of the findings and discusses the implications of the study.

## 2. Main Architecture and Materials

**Figure 2:**
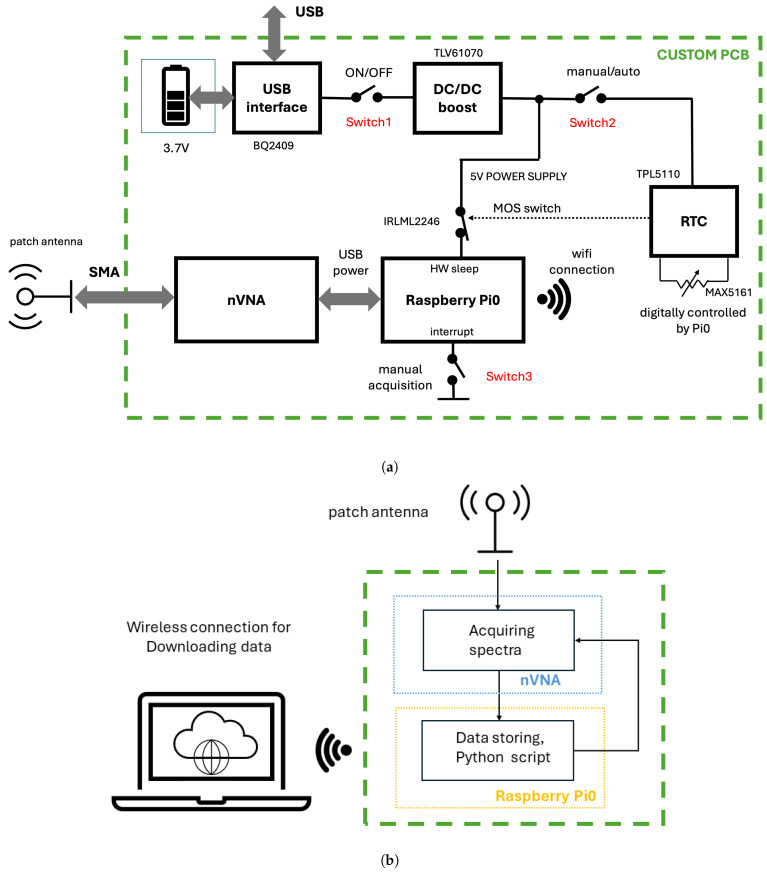
(**a**) Hardware structure of the proposed PCB; (**b**) data flow scheme.

The simplified HW structure is presented in Figure 2a, where the NanoVNA is controlled by a Raspberry Pi to gather spectra from the nanoVNA connected with an external patch antenna. The controls and the power supply of the NanoVNA are delivered through an internal USB connection. The architecture has been conceived to work for two kinds of acquisitions, manual and automatic, using additional components located in a custom PCB. The Raspberry Pi is powered by a DC/DC converter through an MOS switch that is normally set to ON. In the manual mode, the NanoVNA and the Raspberry Pi are always powered, and a single acquisition is enabled by an HW interrupt. In automatic mode, a real-time clock (RTC) is enabled, which can put the Raspberry Pi in HW sleep by switching off its power supply through the MOS transistor. The acquisition timing is set by a digitally controlled resistance connected to the RTC. The data flow is shown in Figure 2b. The overall control is performed by the Raspberry Pi using Python scripts that run in its operating system. All the spectra are temporarily stored in the Raspberry’s internal memory and transferred to an external PC through a Wi-Fi link on demand to be processed offline. Alternatively, in a more advanced implementation, the predictive models could be directly implemented in the Raspberry to achieve direct hydration estimation to realize a fully autonomous sensor. In automatic mode, a script is run during the wake-up phase when the Raspberry is powered on. All the acquisition parameters are previously set in the script during the setup.

### 2.1. The Core HW Components

The overall system is based on the integration of the following two core components:

#### 2.1.1. Raspberry Pi

Raspberry Pi is a series of small SBCs developed in the United Kingdom by the Raspberry Pi Foundation in association with Broadcom [47]. The presented study depends on the Raspberry Pi Zero W model. This new iteration is a further development of the Raspberry Pi Zero, an extremely affordable model that could be purchased at retail prices down to a few dollars upon its release in 2015. This version offers integrated Wi-Fi and Bluetooth low energy (BLE) functionality, which eliminates the need for external adapters and enhances flexibility. This model boasts the following key features: a 1 GHz single-core GPU, 512 MB of RAM, a Mini HDMI port, a micro USB on-the-go (OTG) port, and a HAT-compatible 40-pin header. It runs a 32b Pi OS, a Unix-like operating system based on the Debian GNU/Linux distribution on which Python 3.0 scripts can run, managing data acquisition via the NanoVNA and saving the results as text files.

#### 2.1.2. NanoVNA

NanoVNA V2 (S-A-A-2) is a 4 GHz VNA [48]. Its wide frequency range makes it versatile for various applications, including HF radio, Wi-Fi, and cellular networks. Moreover, the NanoVNA V2 provides low noise and enhanced temperature stability, both crucial for high-precision, long-term measurements. Furthermore, the improved USB protocol and software support enable seamless integration with other systems for real-time data streaming and advanced analysis. Before conducting measurements, a thorough calibration is carried out using an open-short-load protocol following standard procedures to ensure high accuracy. The NanoVNA V2 allows the computation of the complete set of four scattering parameters (S-parameters) in the 50 KHz–4.4 GHz range using two ports. The setup can be selected by the user through the LCD screen. The acquisitions can be conducted by linking the NanoVNA to a computer and employing the specialized software. Alternatively, it can be connected to the Raspberry Pi via a USB cable and utilize a Python function to configure the settings, execute calibration, and obtain the spectrum. When connected to external devices, power is supplied through the USB cable. The used NanoVNA model in the present paper is an unofficial clone called “3.2 black and gold”, but it is compatible with the official software. The primary distinction between the official version and this clone lies in the inclusion of an external battery, enabling its use as an autonomous system. Upon removing the external casing, it is revealed that the NanoVNA comprises two PCB boards. One houses circuits that are essential for the LCD screen and the battery. At the same time, the other one accommodates all components necessary for generating electromagnetic waves and acquiring the T/R waves. To make a spectral acquisition, the NanoVNA needs to be connected to another system. Usually, it is connected to a PC with a USB cable, and acquisitions are performed thanks to a software called NanoVNA-QT, downloadable from its official website.

### 2.2. Custom PCB Design Elements

**Figure 3:**
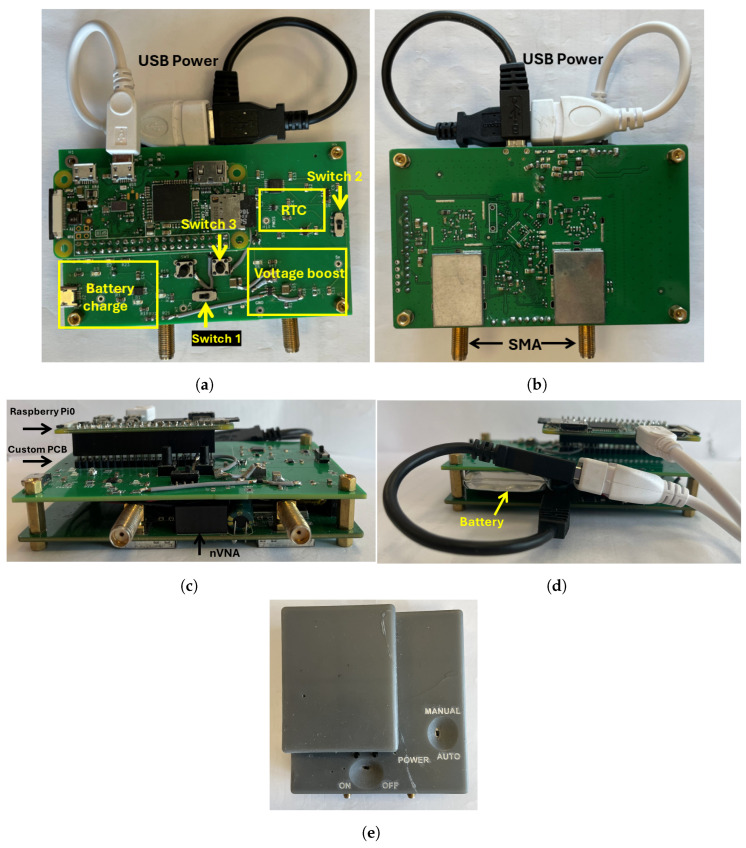
PCB and case implementation: (**a**) front view; (**b**) back view; (**c**) side front view; (**d**) side back view; and (**e**) case made using 3D printing.

A compact PCB has been designed, as depicted in Figure 3, whose purpose is twofold. Firstly, it connects the Raspberry Pi with the VNA and external switches/LEDs. It accommodates the Raspberry Pi using a 20×2 pin header. This configuration ensures that all GPIO pins utilized for our application are electrically connected to the rest of the circuit. Since the NanoVNA screen is not needed for our purposes, we opt to utilize only the second PCB. The PCB we designed will replace the first one and incorporate a new battery. This configuration allows it to be compatible with both the official version of the NanoVNA and our modified setup. The mechanical design facilitates the physical connection between the two devices: it is crafted to be mountable atop the NanoVNA, enabling the Raspberry Pi to be soldered onto it using a 40-pin header. This setup results in a compact and self-sufficient device. Additionally, leveraging the Raspberry Pi’s Wi-Fi capability, the device can be connected to the internet seamlessly. The power-on and -off cycle, combined with a one-spectrum acquisition, takes less than 1.5 min, showcasing the system’s efficient operation. Despite its single-core CPU, the prediction process is swift: experimental trials demonstrate that once a spectrum is acquired, the entire computation takes approximately 1 min. Secondly, it hosts other components that are used for governing the system, specifically:

1 A USB interface chip BQ2409 (Texas Instruments, Dallas, TX, USA): This serves to connect the system with a 3.7 V battery. It facilitates charging the battery using standard power cables (similar to the one utilized for the Raspberry Pi) via the micro USB port, even while the system is operational.
2 A step-up DC/DC converter TLV61070a (Texas Instruments, Dallas, TX, USA): It converts the 3.7 V output from the battery to 5 V for powering both the Raspberry Pi and the RTC.
3 An RTC TPL5110 (Texas Instruments, Dallas, TX, USA): It is used for the time control of the system in automatic acquisition. It is linked to a digital potentiometer (MAX5161 (Maxim Integrated Products, Sunnyvale, CA, USA) [49]. Additionally, the real time clock (RTC) is connected to a PMOS transistor (Infineon IRLML2246 (International IoR Rectifier, CA, USA)) [50], which is responsible for controlling the 5 V line. This control allows for a complete deep sleep of the Raspberry Pi when it is not in use.
4 Temperature and humidity sensors SHT40 (Sensirion, Staefa ZH, Switzerland): This sensor is used for monitoring both internal and external temperature and humidity, with humidity accuracy of ±1.8% RH and temperature accuracy of ±0.2 °C. It is directly connected to the Raspberry Pi via the I2C protocol.

As illustrated in Figure 3, there are switches included to power up the system and enable manual data acquisition. Switch 1 is specifically employed to power up the Raspberry Pi and the other associated components. Conversely, Switch 2 is designated to power only the RTC. This system enables the implementation of two modes of operation. In the manual mode, the Raspberry Pi remains powered on until it manually shuts down, allowing for single spectra acquisition by pressing Switch 3. Conversely, in the automatic mode, an RTC module, the TPL5110, is utilized [51], which drives a MOSFET that is connected to the power via the Raspberry Pi. With this, it is possible to turn on the Raspberry Pi automatically only for the time necessary to perform and save an automatic acquisition, greatly enhancing the duration of a single battery charge. A second manual Switch 2 allows one to turn on the RTC only when the automatic acquisition mode is required. The period between two acquisitions is defined by the value of the resistor connected to the RTC. The digital potentiometer MAX5161, by Maxim Integrated, is used, which can take on 31 different resistance values, ranging from 0 to 200 kΩ. This configuration empowers the user to select the period between two consecutive acquisitions via the Raspberry Pi before initiating the acquisition phase. Two level shifters [52,53] are used to facilitate communication between the Raspberry Pi, which features 3.7 V output pins, the RTC, and the digital potentiometer, which operates with 5 V.

#### Battery Management

This system is primarily executed by the TLV61070a. It takes the 3.7 V battery as input and provides a 5 V output for powering the Raspberry Pi and all other components. Additionally, it facilitates battery charging via a micro USB port. Lastly, four LEDs visually indicate the battery charge status. When the battery charge is above 75%, all four LEDs are illuminated, gradually decreasing to just one LED when the charge falls below 25%. The entire system is powered on/off using a manual switch, Switch 1, which is positioned between the battery connector and the TLV61070a.

### 2.3. External Patch Antenna

**Figure 4:**
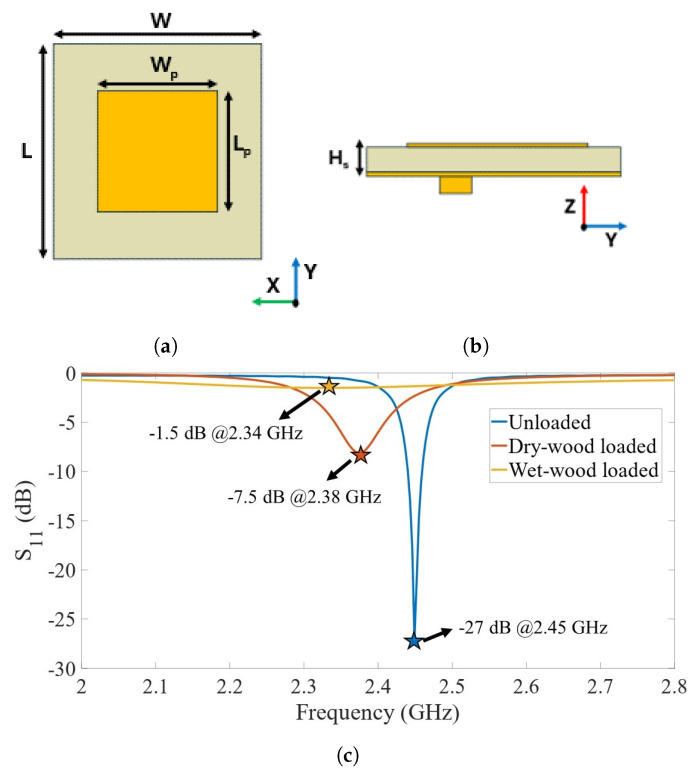
(**a**) Top view; (**b**) lateral view of the proposed antenna with its dimensions (W=L= 50 mm, $W_{p}=L_{p}=24.4$ mm, and $H_{s}=0.61$ mm); and (**c**) simulated reflection coefficient in three different loading conditions: free-space scenario, dry wood loaded, and wet wood loaded.

The sensing element is a patch antenna operating at 2.45 GHz, realized on a typical RF substrate Rogers RO4360G2 (Rogers corporation, Evergem, Belgium) with $\epsilon_{r}=6.15$, 0.610 mm substrate thickness, and electrodeposited copper with 35 µm thickness). A coaxial feeding is chosen for the antenna, to avoid direct contact of the feeding section with the wood sample. The antenna is firstly simulated through full-wave electromagnetic (EM) simulation, in a CST STUDIO environment. Figure 4a,b show the top and lateral views of the proposed antenna, respectively. The total dimensions of the radiating element are 50×50 mm^2^. A superstrate, when placed above a patch antenna, significantly influences the reflection coefficient by altering the impedance matching between the antenna and the surrounding medium. This happens because the superstrate modifies the effective dielectric constant around the patch, leading to changes in the resonance frequency and bandwidth of the antenna. For this reason, EM simulations are carried out to predict the behavior of the antenna in three distinct cases: unloaded antenna (free-space condition), dry wood loaded, and wet wood loaded. The electrical properties of the wood in the S-band are extracted from [54].

The antenna is firstly optimized in the free-space scenario, where a specification of a reflection coefficient lower than −20 dB is set as a requirement. The antenna is then simulated in the loaded condition by the wood sample presenting different MC levels. To reproduce the laboratory measurement conditions, the dimensions of the wood sample inserted in the EM simulations are the same as those of the real one.

Figure 4c shows the simulated reflection coefficient of the patch antenna in the unloaded, fully-dried-wood-loaded, and fully-wet-wood-loaded condition, in the 2–2.8 GHz band. As expected, the predicted S_11_ strongly depends on the superstrate medium and its electrical properties. From the free-space scenario, where the antenna presents a minimum peak at 2.45 GHz of −27 dB, the deviation in terms of the center frequency is 70 MHz and 110 MHz for the dry-wood-loaded and wet-wood-loaded antenna, respectively. A strong change in the minimum value of the S_11_ is also observed. For the dry-wood-loaded antenna, a −7.5 dB peak is observed, whereas for the wood-loaded patch, it is only −1.5 dB.

The high penetration depth of the electric field strength produced by the patch antenna is verified by monitoring the electric field along the wood sample section employing full-wave simulations. The wet wood is used as a test case. In particular, for a trunk sample of dimensions $19.0\times 20.5\times 20.7\;{\mathrm{cm}}^{3}$ (longitudinal × radial × tangential, L × R × T), a 25% decrease in the electric field strength is observed after 15 mm of the patch-wood transition.

## 3. Modes of Operation

**Figure 5:**
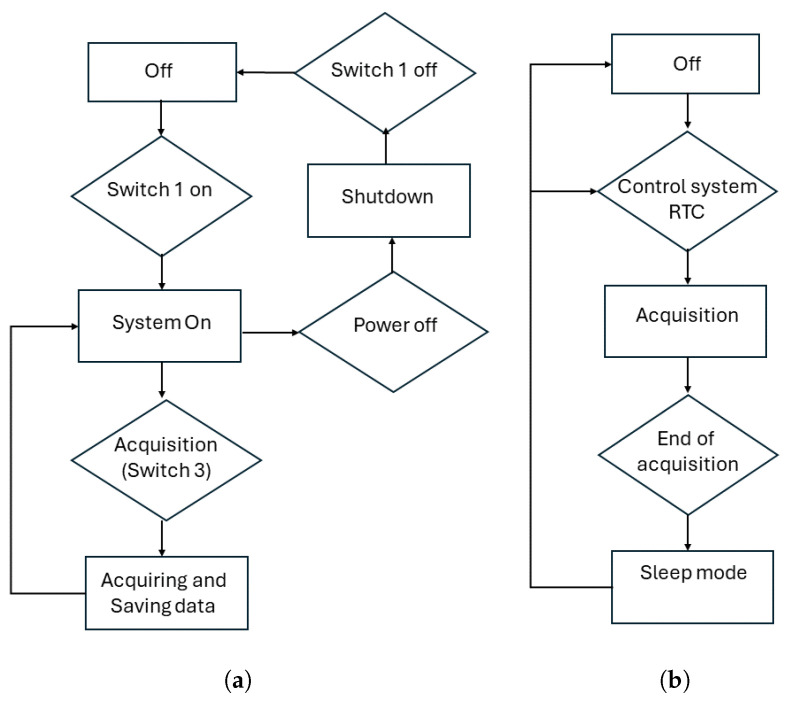
Mode of acquisitions: (**a**) *manual*; (**b**) *automatic*.

Acquisition of the spectral data with the NanoVNA using a Raspberry Pi is performed by a Python script that runs in the Raspberry operating system. As previously mentioned, the existing control system integrated into the PCB enables toggling between two acquisition methods as presented in Figure 5. The first mode is labeled as manual, while the option for automatic can be engaged using Switch 2.

*Manual mode:* The system is activated using Switch 1 after a wait period approximately less than one minute. A brief press of Switch 3 enables spectrum acquisition. Each spectrum acquisition takes approximately 10 s. To conclude the acquisition session, the user must press and hold Switch 3 for more than 1 s to initiate the shutdown of the Raspberry Pi. After waiting approximately 5 s, the power can be turned off using Switch 1.

*Automatic mode:* Upon activating the system with Switch 1, the user is required to establish a connection with the Raspberry Pi and specify the interval between acquisitions by executing a Python function from the command line. Following this setup, the user must shut down the Raspberry Pi and then activate the RTC using Switch 2. Subsequently, the system will automatically power up the Raspberry Pi, which will proceed to autonomously acquire a spectrum before shutting down. This action prompts the RTC to deactivate the 5 V power. To halt the automatic acquisition process, simply power off the entire system using Switch 1 and then toggle Switch 2 to disable automatic acquisition. In summary, spectra will be continuously acquired and stored in the Raspberry Pi’s memory until the *automatic* mode is halted by toggling the secondary power switch, Switch 1.

## 4. Case Study: Greenwood Moisture Content Detection Using a Patch RF Antenna

The application is addressed by acquiring multiple spectra in a dataset with known MC (determined by the oven-dry method). Subsequently, a predictive model would be developed offline and embedded in the proposed PCB. This embedded model could be enabled in real time for detecting the MC in wood, as the sensor acquires spectra in its operation mode. The proposed measurement system operating within the frequency range of 2.2–2.5 GHz was selected to estimate the MC of a silver fir tree trunk chop. The antenna is affixed to the central area of a flat, horizontal surface of the greenwood sample.

### 4.1. Experimental Setup

**Figure 6:**
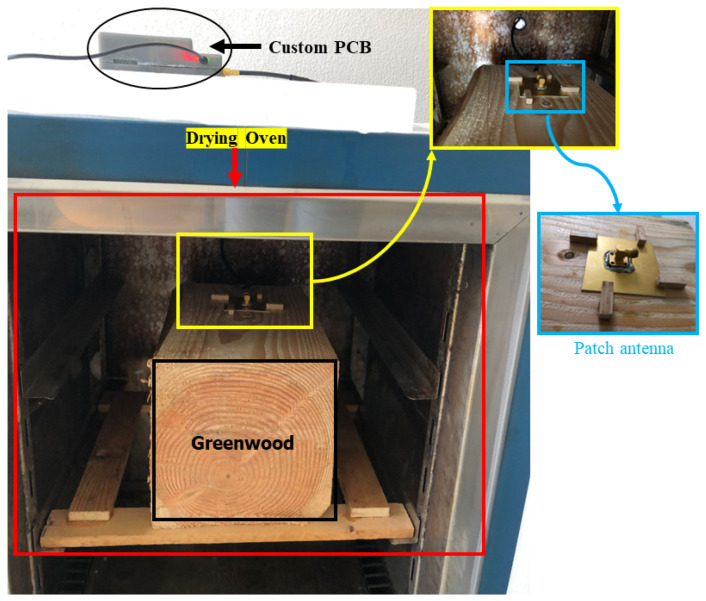
Experimental setup comprising a patch antenna, a drying oven, a greenwood sample, and the customized PCB.

Measurements were conducted on silver fir (Abies alba) greenwood chops with a size of around 19.0×20.5×20.7 ${\mathrm{cm}}^{3}$ (longitudinal × radial × tangential, L × R × T), as seen in Figure 6. They were freshly placed in a drying oven with a controlled temperature set at 40 °C, attached to a patch antenna, the latter connected to the PCB. The initial MC of the greenwood in this study measured by the oven-drying method is 110%. Greenwood is commonly described as recently cut timber where the cell walls are fully soaked with water, and there might be extra water within the cells. The moisture level in green wood typically varies from 30% to over 200% [55]. The target parameter (MC, on a dry basis (%)) used for the statistical inference was obtained by the traditional weighting method, in which it was weighted multiple times a day. For a given piece of wood, the MC can be calculated as [56]:

(1) $$MC\left(\%\right)=\frac{m_{\mathrm{greenwood}}-m_{\mathrm{ovendry}}}{m_{\mathrm{ovendry}}}\times 100$$

**Figure 7:**
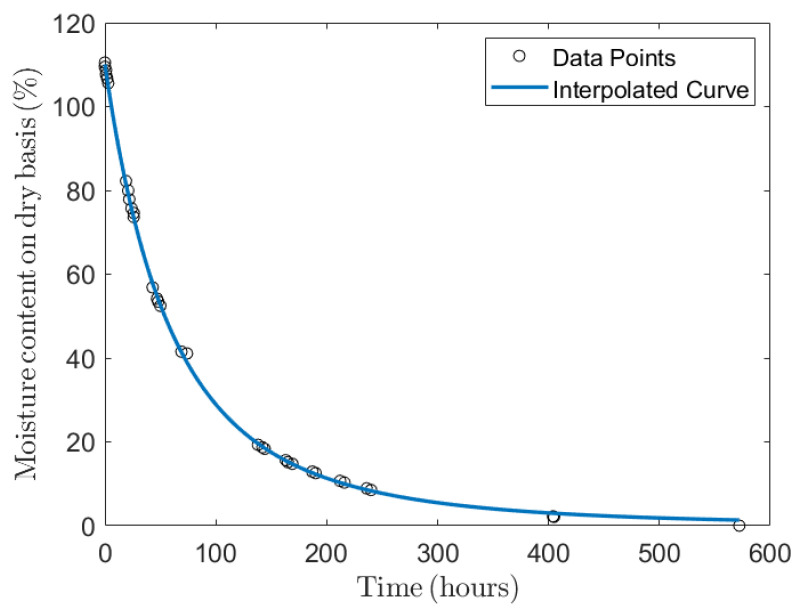
Drying curve of the greenwood on a dry basis vs. time.

$m_{\mathrm{greenwood}}$ is the mass of the specimen at a given MC in kg, and $m_{\mathrm{ovendry}}$ is the mass of the oven-dry specimen in kg. The specimen initially weighed 6.944 kg, but by the end of the drying period, its weight had decreased to 3.299 kg. This weight reduction took place between the 4 June and the 28 June 2024. As illustrated in Figure 7, the recorded MC values over this period closely follow a model that has been previously documented in the literature [57]. The decision to halt the drying process was based on the observation that there were changes below the resolution of the instruments in the $S_{11}$ and the weight of the specimen. We collected 33 MC experimental data points from the 4th to the 28th. Unlike spectra acquisitions, which are acquired automatically by the system, weight measurements were taken by hand. Therefore, the number of collected spectra is much greater than that of the weight measurements. Hence, to make a predictive model, we need to associate each spectrum with a weight (i.e., hydration status). To do so, we used the fitting model shown in Equation (2), from which 911 points were extrapolated to be associated with spectra. The equation is a function of the time *t* (expressed in hours) up to 572 h, which corresponds to the experimental duration.

(2) $$f\left(t\right)=A{\left(1+B\times t\right)}^{-C}$$

where f(t) is the MC percentage at time *t*, A=110, $B=5.2\times 10^{-3}$, and C=3.19.

### 4.2. Statistical Analysis

**Figure 8:**
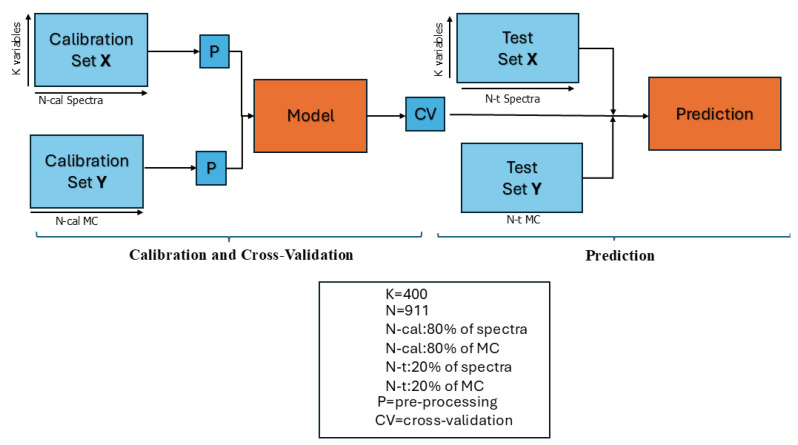
PLSR scheme for calibration, CV, and test validation.

A regression approach was chosen to construct the models from the $S_{11}$ data (the spectral variables). These spectra served as independent variables and were arranged into a matrix X (N×K), also referred to as a dataset matrix, containing *N* acquired spectra, each defined by the *K* frequencies variables. Furthermore, for developing predictive models, we collected MC into a Y(N×1) output vector. This was achieved using a bilinear regression technique known as partial least squares regression (PLSR) analysis. First, the dataset matrix is “well represented” by a matrix of the same size $\hat{X}=TP^{T}$, where T is (N×A), referred to as the score matrix, and $P^{T}$ is (A×K), called the loadings matrix. In other words, $\hat{X}$ is a projection of X in a subspace of A<K orthogonal variables of maximum variance directions. Then, we represent an estimate of the output along the same direction **$\hat{Y}$** as

(3) $$\hat{Y}=TQ^{T}+F=\hat{X}PQ^{T}+F,$$

where Q and F are the loading and error matrices of **Y**, respectively [58,59]. PLSR finds the *A* orthogonal direction to maximize the covariance between input and output in the newly defined spaces. Arranging Equation (3), we have the linear correlation expression

(4) $$\hat{Y}=\hat{X}\beta +\beta_{0}$$

where β is a (K×1) coefficient array. The latter relationship embeds the predictive model. In operating mode, newly acquired spectra are treated with Equation (4) to estimate the moisture percent. The experimental plan included 911 measurements across 400 frequency points within the range of 2.2–2.5 GHz within 572 h. The independent variables in the X dataset were organized in a matrix with dimensions N=911 (spectra measurements) × K=400 (frequency points) for the $S_{11}$ parameter, and N=911 (spectra numerical derivative) × K=399 (frequency points), and the dependent variable Y (dataset MCs) was arranged as (911 × 1). Spectral sample outliers were identified before initiating the model-building process on the complete dataset. Cross-validation [60] and test set validations were subsequently conducted to assess how the models performed with unfamiliar samples to test the model’s prediction ability without needing other acquisitions, as illustrated in Figure 8.

The model’s ability to estimate wood MC was evaluated using several metrics: the coefficient of determination ($R^{2}$), root mean square error (RMSE), significant PLSR components (latent variable (LVs)), and bias. The $R^{2}$ describes the correlation capability of the model, and when it is close to 1, it indicates a high correlation between the model’s input and output. However, the parameter RMSE is preferred to evaluate the efficacy of a predictive model using the cross-validation (CV) approach [61]. Finally, bias refers to the average difference between the model’s estimates and the actual measured values. It indicates whether the model consistently underestimates or overestimates the true values. Usually, preprocessed spectra are used in place of spectral raw data, and based on the RMSE parameter, we found that using the derivative of the spectra is one of the most effective and computationally simple preprocessing approaches. For the test set validation, 20% of the samples were randomly extracted from the calibration dataset, which comprised 80% of the samples, and used to validate the model. The procedure was replicated many times, each time using a different random test set, and subsequently, the results were averaged.

### 4.3. Results and Discussion

**Figure 9:**
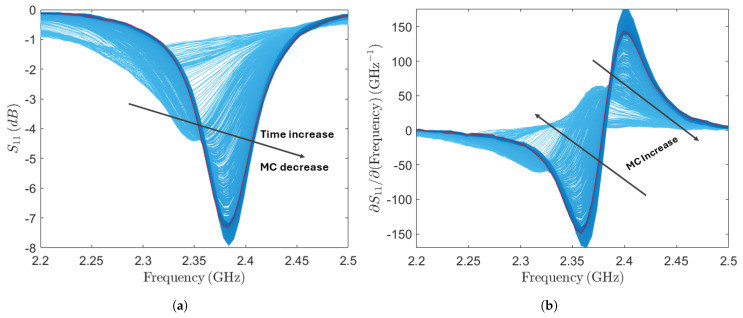
(**a**) Magnitude of $S_{11}$; (**b**) its derivative highlights differences in spectra due to MC change.

In Figure 9, the S_11_ and its derivative results for the greenwood sample are depicted. These components are color-coded to highlight variations in MC. The frequency range spans from 2.2 to 2.5 GHz, corresponding to the region of interest. Measurements, carried out using the nanoVNA, report a good agreement with simulations. In particular, for the dry-wood-loaded patch antenna, the resonant frequency is about 2.38 GHz, and the minimum peak corresponds to about −8 dB of the reflection coefficient at the same frequency. As noticed, fluctuations in MC result in changes in spectral characteristics, including alterations in the intensity of the peak and shifts in resonance frequencies. The dielectric properties of wood, which vary with MC, significantly impact the propagation of electromagnetic waves. Potentially wet wood has a higher dielectric constant due to its water content, leading to greater absorption and rapid attenuation of the waves, resulting in lower-intensity signal peaks [62]. Conversely, with lower MC, dry wood exhibits reduced absorption and attenuation, producing longer and higher-intensity signal peaks [63,64,65,66]. When wood is wet, the increased dielectric constant shifts the natural resonance frequency of the material. This is due to more free water molecules within the wood structure that can easily polarize in response to the electric field, changing the wood’s overall electric permittivity and resonance frequency. On the other hand, the loss tangent, which represents the ratio between the imaginary (related to energy loss) and real (related to energy storage) parts of the complex dielectric constant is also affected by moisture content. As moisture content increases, the loss tangent typically rises, indicating that the wood becomes more lossy. This higher loss tangent at elevated moisture levels corresponds to greater energy dissipation, further contributing to the attenuation of EM waves and the reduction in signal intensity. Conversely, dry wood’s reduced water content causes a different interaction with electromagnetic waves, maintaining higher frequencies longer due to lesser polarization effects and maintaining a steadier resonance frequency without a significant shift [67]. The model’s accuracy relies on using a representative calibration dataset, covering the full moisture range, from wet to dry wood. Validation, essential for ensuring the model’s future reliability, is performed through cross-validation and test set validation. These methods produce prediction RMSE to gauge model performance. Additionally, bias, another key performance measure, reflects the average difference between predicted and actual values, helping identify any systematic differences between the training and validation sets. The results, including $R^{2}$, RMSE, LVs, and bias, for the calibrated and validated PLSR regression models derived from the $S_{11}$ and its derivative are summarized in Table 1.

Table 1 presents the regression results, focusing on the analysis of the predictive model parameters of $S_{11}$ and its derivative with respect to frequency across the different validation phases: calibration, CV, and test.

**Figure 10:**
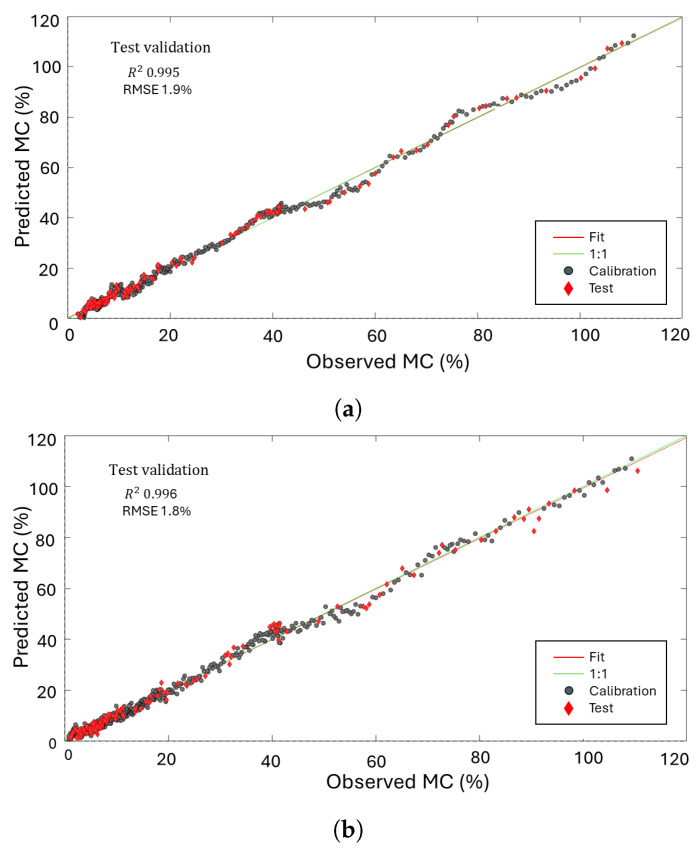
Predicted versus observed MC (in %) for test and calibration of PLSR

**Figure 11:**
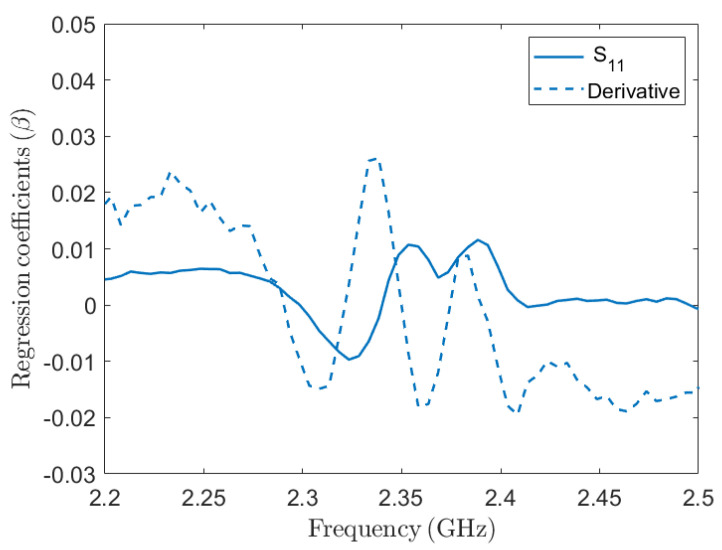
Regression coefficient β for $S_{11}$ and its derivative.

During the test phase, the RMSE for $S_{11}$ is approximately 1.9%, while $R^{2}=0.995$ and the bias is $5.62\times 10^{-5}$, indicating a strong correlation and minimal bias. For the derivative of $S_{11}$, the test phase, the RMSE decreases to 1.8%, while the $R^{2}$ increases to 0.996, and the bias is $-6.60\times 10^{-5}$. These results show a good performance in predicting the MC over the range of (110–0)% for both the $S_{11}$ and its derivative. When comparing our findings to the existing literature, such as [68] on NIR spectroscopy for Korean Pine moisture content, we observe similar success in utilizing PLSR to model complex spectral data. However, where NIR techniques showed reduced accuracy for moisture content above 30%, our microwave-based approach maintains reliable predictions across a broader moisture range. This can be attributed to the deeper penetration capabilities of microwave sensing, which makes it more suitable for high-moisture-content scenarios. Figure 10 illustrates the comparison between predicted and observed MC values for the $S_{11}$ parameter and its derivative. It provides a visual representation of how well the PLSR model aligns with MC measurements under test validation and calibration conditions. The inclusion of derivative analysis serves the purpose of enhancing the prediction accuracy, as evidenced by comparing the RMSE. While these metrics provide a quantitative assessment, a clearer understanding emerges from visualizing the observed versus predicted MC. This approach rectified these nuances, leading to improved model performance and more accurate and linear predictions, as demonstrated in Figure 10. Then, regression β-coefficients are calculated to express a linear combination involving both observed and predicted values, based on the $S_{11}$ parameter and its derivative models, as illustrated in Figure 11. Therefore, it shows which segment of the spectra primarily accounts for explaining variability in the data. For both $S_{11}$ and its derivative, the maximum variation of the regression β is around 2.27–2.45 GHz, which is the most sensitive range for the patch antenna. This analysis demonstrates that our model effectively captures the key spectral features within this critical frequency range. The substantial variation of β-coefficients in this range supports the performance of our model in explaining the variability observed in the data. Regarding model validity, the concentration of β-coefficient variation in the 2.27–2.45 GHz range aligns with theoretical expectations and the sensitivity of the patch antenna. This alignment confirms that our model is robust and accurately represents the relationships within the spectral data. After extracting the regression coefficients β (399×1), it can be stored in the PCB. This allows for the straightforward acquisition of spectra and direct calculation of MC through simple mathematical operations using Equation (4).

Additionally, beyond agricultural applications, this system demonstrates significant potential for adaptation to other biological and environmental monitoring tasks. For instance, the same system initially presented for wood hydration monitoring is modified for use in skin hydration monitoring with minimal changes. By replacing the patch antenna with a CSRR, the system is used to monitor hydration levels in human skin [45]. In this related work, the system, coupled with the CSRR, was used to successfully monitor skin hydration across different body regions, demonstrating its versatility and adaptability. This capability highlights the potential for the system to be employed in broader biological applications, as well as other environmental monitoring tasks, simply by swapping the sensor while retaining the core measurement and data processing components.

## 5. Conclusions

This paper presented a novel system for monitoring wood MC that integrates hardware and ML techniques to improve the efficiency of agricultural practices. The main contributions of this work are twofold. From an architectural standpoint, we developed a compact and affordable system that integrates a Raspberry Pi Zero W and a NanoVNA V2 (S-A-A-2) operating in the 50 KHz–4.4 GHz range, alongside a custom-designed PCB. We also introduced a robust data acquisition procedure using Python scripts for both manual and automated S-parameter acquisition and data storage. From a case study perspective, the system was successfully coupled with a patch antenna to assess the hydration of greenwood. Monitoring the MC of wood is vital for assessing tree health and optimizing irrigation practices to enhance agricultural efficiency. Therefore, the availability of cost-effective and dependable devices is paramount. Microwave techniques offer a promising approach for accurately measuring wood moisture, particularly by analyzing the scattering parameter ($S_{11}$). A stand-alone method utilizing a patch antenna coupled with a custom PCB has been developed for direct measurement of MC in fresh wood. At present, economic viability is crucial for widespread adoption, and the present device effectively meets this requirement. The $S_{11}$ spectra acquired at 2.2–2.5 GHz on greenwood and its derivative, capturing different MC from 110% to 0% (dry basis), were used to build PLSR models. Both models are accurate in predicting MC. However, the derivative spectra model offers a slight advantage, with $R^{2}=0.996\;\mathrm{and}\;\mathrm{RMSE}=1.8\%$, which slightly improves the model linearity. The original spectra also perform well with a $R^{2}=0.995\;\mathrm{and}\;\mathrm{RMSE}$ = 1.9%. As expected, variations in wood temperature can affect the spectral waveforms of the *S*_11_ parameters. However, the multivariate analysis indicated that MC was the most influential variable contributing to the variance of the waveforms. The optimal operating bandwidth was identified to be between 2.27 and 2.45 GHz. This limited bandwidth contributes positively to the affordability of the device. Additionally, the integration of the new PCB played a crucial role in achieving these promising results, facilitating accurate predictions of MC.

## 6. Future Work

In future work, we aim to extend our validation by comparing the proposed technique with other existing techniques for measuring trunk hydration and by including a broader range of environmental conditions and wood types. This will include experiments conducted across varying temperature and humidity levels, along with different wood species, to assess the sensor system’s robustness and adaptability.

Additionally, recognizing the importance of rigorous validation, we plan to incorporate comprehensive statistical analyses in our upcoming studies to further ensure the accuracy and reliability of the model’s predictions.

## Figures and Tables

**Table 1:** Results of PLSR parameters for $S_{11}$ and its derivative across various validation processes.

	Process	RMSE (%)	$R^{2}$	LVs	Bias
	Calibration	1.7			$-5.51\times 10^{-17}$
S_11_	CV	1.8	0.995	6	$5.62\times 10^{-5}$
	Test	1.9			$5.62\times 10^{-5}$
	Calibration	1.8			0
$\partial S_{11}/\partial \;\left(\mathrm{Frequency}\right)$	CV	2.0	0.996	6	0
	Test	1.8			$-6.60\times 10^{-5}$

## Data Availability

Dataset available on request from the authors.
